# Immunoglobulin G4-related Disease: Presentation of the First Case with Isolated Pterygopalatine Fossa Involvement

**DOI:** 10.7759/cureus.4719

**Published:** 2019-05-22

**Authors:** Georgios P Kotsis, Aikaterini P Peteinaki, Athanasios C Sakellaridis, Efthymios E Andriotis, Nektarios Koufopoulos

**Affiliations:** 1 Otorhinolaryngology / Head & Neck Surgery, “Saint Savvas” General Anti-Cancer and Oncological Hospital of Athens, Athens, GRC; 2 Miscellaneous, School of Medicine, University of Thessaly, Athens, GRC; 3 Interventional Radiology, “Saint Savvas” General Anti-Cancer and Oncological Hospital of Athens, Athens, GRC; 4 Pathology, Attikon University Hospital, Medical School of Athens, Athens, GRC

**Keywords:** head and neck, igg4, igg4-related disease, pterygopalatine fossa

## Abstract

Immunoglobulin G4-related disease is an immune-mediated fibroinflammatory disease with single or multiple organ involvement. Clinically it mimics several benign and malignant tumors, as well as infectious, and inflammatory disorders. It typically presents as multiple tumor-forming lesions. Histological and immunohistochemical findings are characteristic. Serum immunoglobulin G4 levels are usually increased. Systemic corticosteroid administration is the treatment of choice with good response, especially in early disease stages.

We present the first case of immunoglobulin G4-related disease presenting as an isolated tumor forming lesion of the left pterygopalatine fossa. Imaging studies indicated a benign process. Histological findings were consistent with IgG4-related disease. The patient showed a good response to systemic corticosteroid treatment and remains free of symptoms 18 months following diagnosis.

## Introduction

Immunoglobulin G4-related disease (IgG4RD) is a recently recognized immune-mediated fibroinflammatory disease affecting multiple organs. Clinically it mimics a considerable number of malignant, infectious, and inflammatory disorders [1]. Typically, it presents as multiple tumor-forming lesions, it has characteristic histological findings and often but not always increased serum immunoglobulin G4 (IgG4) levels. Its response to glucocorticoid treatment is excellent and rapid [2].

We present a rare case of IgG4RD presenting as a tumefaction of the left pterygopalatine fossa. To our knowledge isolated pterygopalatine fossa involvement has not been previously reported in the English literature to date.

## Case presentation

A 66-year-old man was admitted to the head and neck department of our hospital because of recurrent penetrating temporo-occipital headaches mostly located on the left side of the head. Headaches started five years ago which were relieved with paracetamol and/or nonsteroid anti-inflammatory medication per os. The patient referred to our hospital because the episodes of headache had become more frequent and aggravating during the last three months.

**Figure 1:**
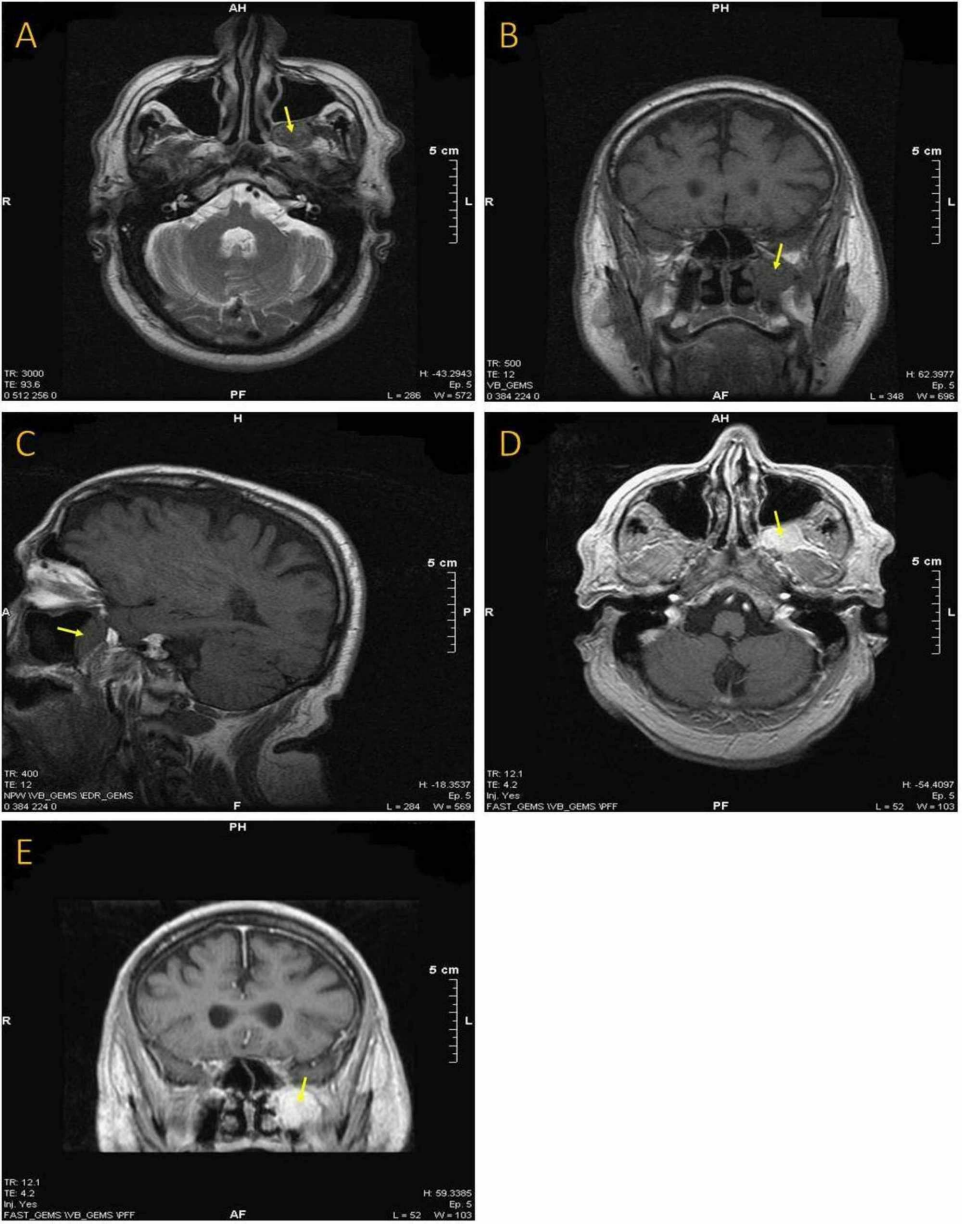
(A) An axial T2-weighted with fat suppression MRI scan showing the lesion located in the left pterygopalatine fossa. (B) A coronal T1-weighted MRI showing the sizeable mass in the same patient. (C) A sagittal T1-weighted MRI demonstrating the lesion located just behind the posterior wall of the left maxillary sinus. (D) and (E) Axial and coronal T1-weighted MRI with gadolinium.

**Figure 2:**
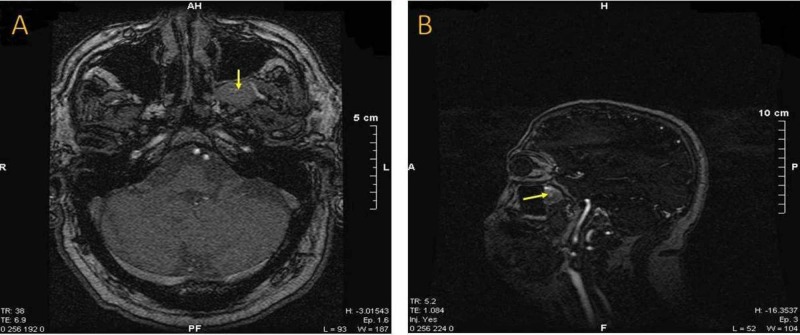
(A) and (B) Magnetic resonance angiography showing lack of vascularization.

**Figure 3:**
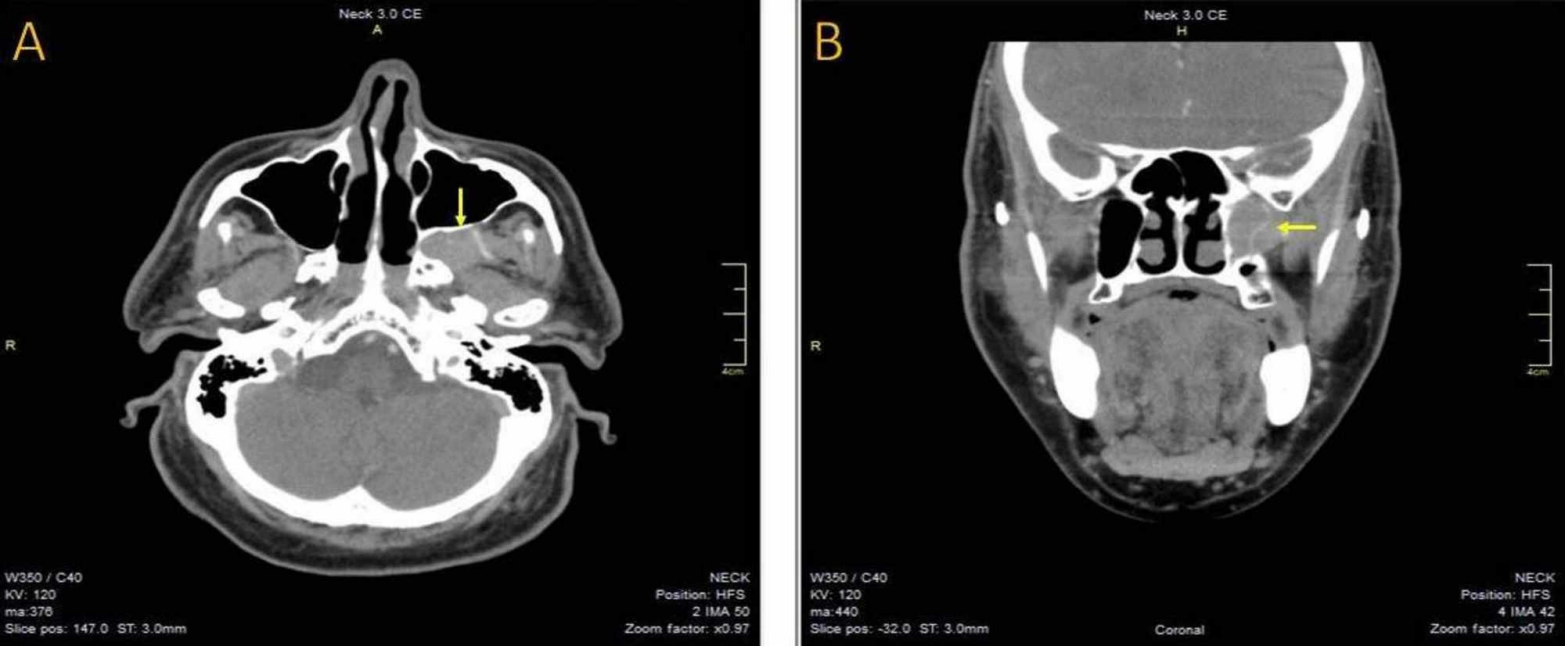
(A) and (B) The axial and coronal views of a CT scan of the sinuses demonstrating an imprint on the posterior wall of the maxillary sinus due to compression.

**Figure 4:**
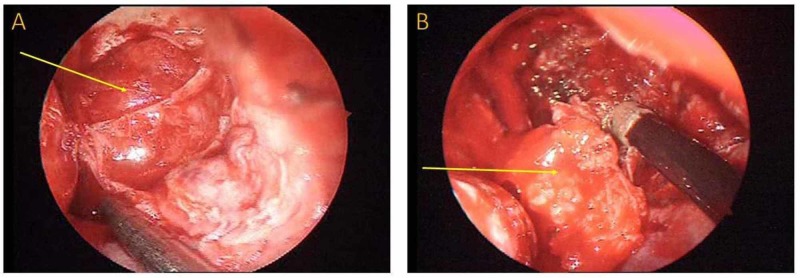
(A) The endoscopic close-up view of the lesion after removing the posterior wall of the maxillary sinus. (B) The segmental endoscopic resection of the mass.

**Figure 5:**
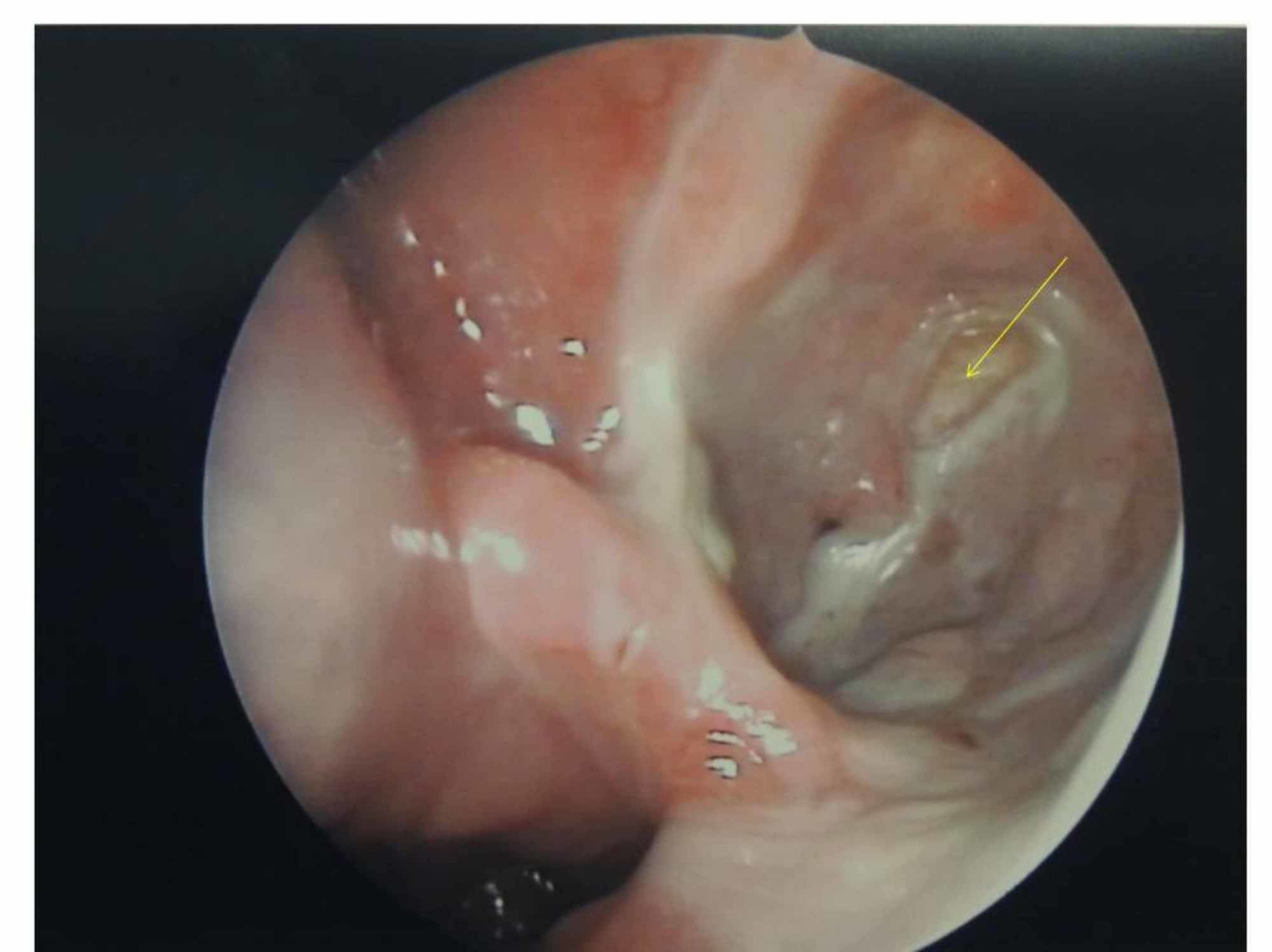
Postoperative endoscopic view of the left maxillary sinus a month later.

MRI revealed a left pterygopalatine fossa sizeable mass-forming lesion (Figure 1A-1E). Magnetic resonance angiography showed lack of neovascularization without any other abnormal finding, indicating a potential benign lesion (Figure 2A, 2B). Subsequently, the patient was admitted to the head and neck department. A CT scan of the sinuses was performed, demonstrating an imprint on the posterior wall of the maxillary sinus due to compression. There was no bone erosion, a second indication that the lesion was not malignant. In addition to these radiological studies, high definition CT scan and three-dimensional (3D) reconstruction were performed to assess the exact location of the mass and its relationship to the surrounding structures. The latter showed that the medial maxillary artery was crossing the tumor (Figure 3A, 3B). All the routine preoperative exams were normal. The segmental removal of the lesion along with its surrounding tissue was achieved through an endoscopic transnasal approach to the pterygopalatine fossa (Figure 4A, 4B). The mass then was sent for a frozen section biopsy which was negative for malignancy. Ligation of the medial maxillary artery was performed due to intraoperative bleeding, followed by a maxillary sinus and anterior nasal packing at the end of the procedure. The package was removed after three days without any postoperative bleeding, and the patient was discharged from the hospital on the fifth postoperative day with no further complications. One month after surgery, the healing process of the posterior wall of the left maxillary sinus was almost completed (Figure 5).

**Figure 6:**
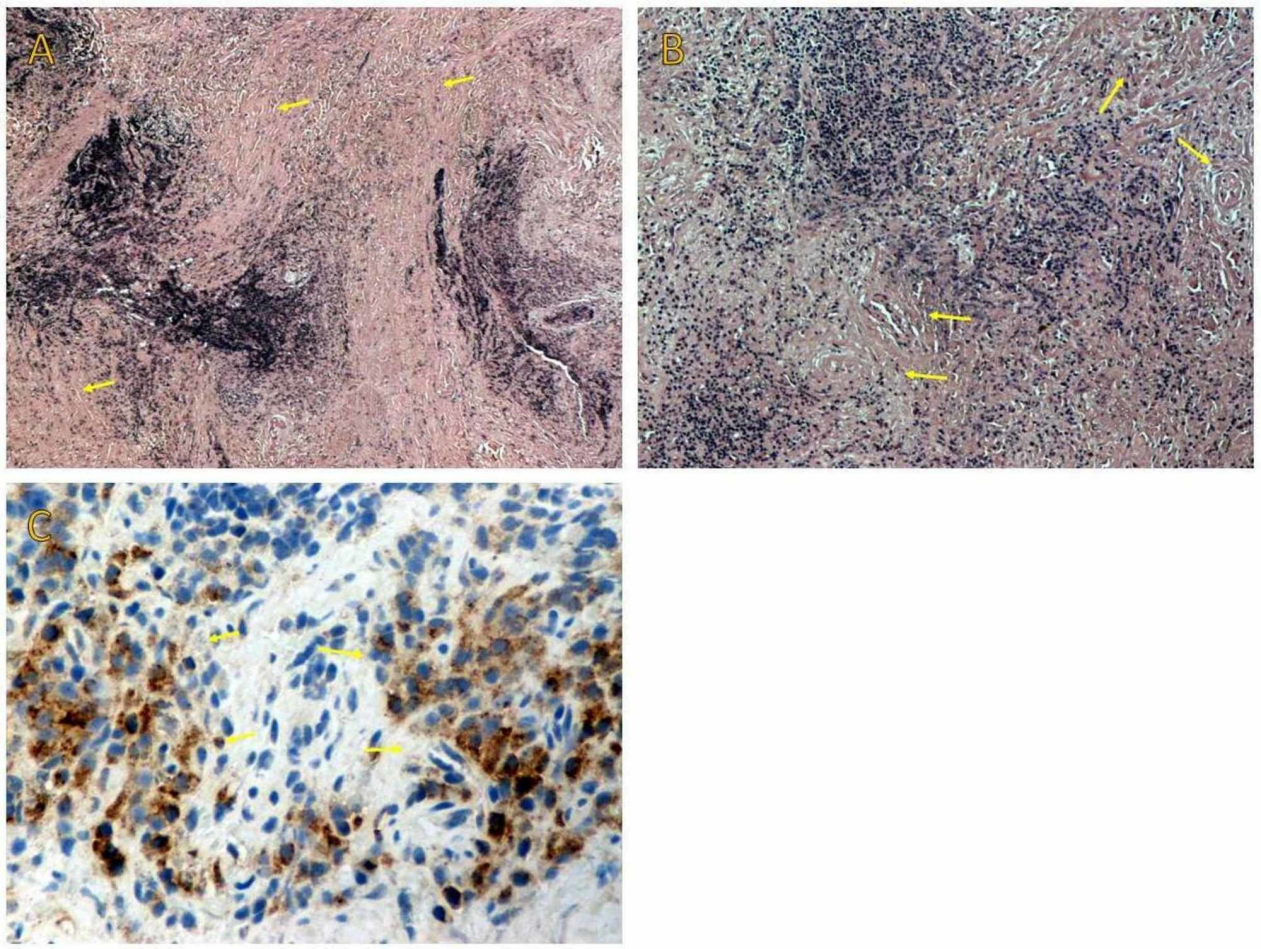
(A) On low power examination, storiform fibrosis and a dense lymphoplasmacytic infiltration are recognized. (B) Obliterative phlebitis is shown on medium power examination. (C) Immunohistochemical stains show infiltration by IgG4-positive plasma cells and a high IgG4 to IgG positive cell ratio. IgG4: Immunoglobulin G4

Microscopic examination of the lesion revealed the existence of storiform fibrosis (Figure 6A), obliterative phlebitis (Figure 6B) and diffuse dense lymphoplasmacytic infiltrations forming several lymphoid follicles. A small number of eosinophils were also present. The immunohistochemical study demonstrated abundant IgG4 positive plasma cell infiltration (Figure 6C) and a high IgG4 to IgG ratio. The combination of morphological and immunohistochemical findings established the diagnosis of IgG4RD.

**Figure 7:**
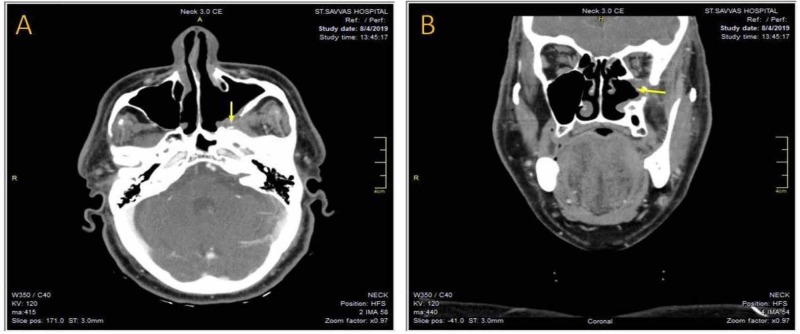
(A) The axial and (B) the coronal views of the postoperative CT scan of the sinuses after 18 months, showing the healed posterior wall of the left maxillary sinus and the left pterygopalatine fossa free of pathology.

Two months after surgery, serum immunofixation test showed an elevation of immunoglobulin G. Serum protein analysis by capillary electrophoresis showed normal levels of Gamma-globulin fraction. A QuantiFeron-TB enzyme-linked immunosorbent assay (ELISA) test was negative thus excluding a Mycobacterium tuberculosis infection. Furthermore, all other routine tests were normal. The patient received orally hydroxychloroquine 200 mg 1 x 2, methylprednisolone 8 mg x 1, calcium 500 mg 1 x 1 and metformin 1000 mg 1 x 2 per day. Eighteen months following surgery the patient shows no signs of recurrence on imaging studies and remains disease-free as evidenced by postoperative CT-scan (Figure 7A, 7B).

## Discussion

IgG4RD is a recently recognized inflammatory disease, first reported in 2003 [1]. In the cases of systemic presentation, it typically affects middle-aged patients with a male predominance (2.8-3.5:1). Regarding the head and neck involvement, there is an equal distribution [1, 2]. The spectrum of IgG4RD encompasses several previously described entities. Its etiology is thought to be autoimmune, inflammatory or allergic, but the exact underlying pathophysiological mechanism still remains unknown [3]. The anatomical structures most frequently affected are the pancreas, salivary glands, lacrimal glands, lymph nodes and retroperitoneum [4]. IgG4RD head and neck involvement is the second most common after pancreas [5]. The organs most commonly affected are the orbit followed by salivary and lacrimal glands and lymph nodes. The thyroid gland, sinonasal cavities, and airways are involved less frequently [2].

The diagnostic approach is difficult since the disease imitates many malignant, infectious and inflammatory disorders and due to lack of clinical suspicion. A combination of clinical, laboratory and histopathological criteria are necessary to make a definitive diagnosis [3]. Clinically patients present with single or multiple organ enlargement [1, 6]. Mild loss of weight and fatigue may be related to systemic disease [7]. Elevated serum IgG4 is found in about half of the patients with IgG4RD. According to the literature, normal serum IgG4 concentrations are found in 3-40% of patients, even in those with a histologically confirmed IgG4RD [3]. Patients with single organ involvement have often normal IgG4 serum levels [3, 6]. Higher serum IgG4 levels tend to correlate with multiple organ involvement [8]. Serum IgG4 elevation is useful for screening but not as a single diagnostic marker since several other disorders including multicentric Castleman’s disease, pemphigus, atopic dermatitis, and asthma have elevated level of serum IgG4 [3].

Histopathological examination is the gold standard for the diagnosis. The three main characteristics of IgG4RD are lymphoplasmacytic infiltration, a variable degree of fibrosis forming a characteristic “storiform” pattern and obliterative phlebitis. The first two characteristics are always present, while obliterative phlebitis is variably present depending on the organ and tissue involved [7]. The hallmark of IgG4RD is to find a high number of IgG4 positive plasma cells as well as an IgG4 to IgG ratio higher than 40% and usually more than 70% [1, 6]. In chronic cases where fibrosis predominates lower numbers of IgG4 positive cells may be present [7].

Treatment consists in systemic administration of corticosteroids and other immunosuppressive drugs. In some cases, surgery may be helpful [3]. Response to corticosteroid administration is better in the initial stages of the disease. Relapse may occur in up to 40% of patients [7].

In our case, the mass lesion had to be differentiated from malignant tumors. Since pterygopalatine fossa involvement in IgG4RD is very rare, this entity was not suspected clinically. Imaging studies (radiological tests, CT high resolution, and 3D reconstruction) were in favor of a benign tumor. Finally, microscopic examination, with the appropriate immunostains combined with the clinical and radiological features allowed us to make the diagnosis of an IgG4RD pseudotumor.

## Conclusions

IgG4RD is a poorly understood and under-recognized immune-mediated systemic fibroinflammatory disease. Its main differential diagnosis concerns several malignant, infectious, and inflammatory disorders. Correct diagnosis is essential because it has a benign clinical course and is responsive to steroid therapy.
